# Protective effect of breviscapine in acute pulmonary embolism rats via regulation of MCP‐1 and IL‐13

**DOI:** 10.1111/jcmm.13871

**Published:** 2018-10-24

**Authors:** Zhenkun Li, Hua Lin, Zhaoxing Dong, Xiaoyuan Zhao, Ling Li

**Affiliations:** ^1^ The Second Affiliated Hospital of Kunming Medical University Kunming 650101 Yunnan China; ^2^ Biomedical Engineering Research Center Kunming Medical University Kunming 650500 China

Dear editor,

Pulmonary embolism (PE) is a blockage of an artery in the lungs by a substance that has travelled from elsewhere in the body through the bloodstream,^1^ which results in between 50 000 and 200 000 deaths in United States every year.^2^

Usually, anticoagulant therapy is the mainstay of treatment for a PE. Low‐molecular weight heparin (LMWH) or fondaparinux is administered initially, while warfarin, acenocoumarol, or phenprocoumon therapy is commenced.^3^ However, the most common side effects of LMWH include bleeding, which could be severe or even fatal, allergic reactions, injection site reactions, and increases in liver enzyme activity, usually without symptoms.^4^

**Here**, traditional Chinese medicine could be another useful treatment method for PE. *Erigeron breviscapus* is a native plant species in Yunnan, China. Breviscapine (Bre) is a purified flavonoid extract from this species. Bre has a broad range of pharmacologic effects, including dilation of micro‐blood vessels, reduction in blood viscosity and improvement of the micro‐circulation. Bre also has an antiplatelet, anti‐thrombotic action and can decrease plasma fibrin content and promote fibrinolytic activity.^5^, ^6^, ^7^ In this study, we evaluated the effect of Bre on PE rat model and analysed the potential mechanism with MCP‐1 and IL‐13, which provided a research foundation and theoretical basis for the clinical application of Bre.

**Firstly**, we constructed model animals. The acute pulmonary embolism (APE) models were constructed with two different kinds of embol (DVT and polystyrene (PS) microspheres respectively) through the same surgical procedure, according to Watts.^8^ And 40 experiment male SD rats (10 weeks old) were involved. After construction of the APE model with DVT and PS, the symptoms were consistent with the clinical manifestations of cardiopulmonary disease, including shortness of breath and diffuse moist rales.

**Then taken grouping and drug intervention.** The Bre drug concentration gradient was as follows: the Bre‐low group was treated with 0.2 mg/kg/day; and the Bre‐high group was treated with 1 mg/kg/day. Bre intervention occurred once every 12 h for 2 days after construction of the APE model. The Bre was purchased from Yunnan Biological Valley Pharmaceutical Co., Ltd. (Kunming, China). All rats had intraperitoneal (i.p.) injections.

The positive control group was treated with LMWH at a dose of 200 IU/kg with same injection schedule, which was purchased from Kunming JiDa Pharmaceutical Co., Ltd. (Kunming, China). The control group was treated with normal saline instead of any drugs.

**Treatment effect as following:** by drug intervention with Bre or LMWH, the clinical symptoms were relieved, as demonstrated by normal eating, drinking and the absence of fainting or twitching. This observation suggested that the drug was effective to some extent. Bre significantly improved these clinical manifestations, especially, in the Bre‐high group through i.p. injection. The rats had moderate improvement in the Bre‐low and LMWH groups.

**Finding of physiological indicators were on the experiment rats.** 0.5 ml of abdominal aortic blood (AAB) and 0.5 ml of peripheral venous blood (PVB) were collected 48 h after drug. Compared with the sham group, the peripheral blood pressure decreased, but the respiratory and heart rates increased significantly in the APE model animals (**Table** 1). The arterial oxygen tension (PaO_2_) was significantly lower and the alveolar‐arterial difference in carbon dioxide tension (PCO_2_) was significantly higher in the APE subgroup than in the sham subgroup (*P *< 0.01).

**Table 1:** The results of physiological indicators for each experiment group

Indicators	Model	Sham	Bre‐low	Bre‐high	LMWH
Respiratory rate (beats/min)	134 ± 5^a^	85 ± 3	117 ± 3^b^	108 ± 4^b^^,^ c	116 ± 4^b^
Heart rate (beats/min)	530 ± 6^a^	371 ± 4	465 ± 5^b^	420 ± 5^#^	440 ± 6^b^
Systolic blood pressure (kPa)	9.03 ± 0.10^a^	13.07 ± 0.03	9.87 ± 0.07^b^	10.96 ± 0.06^b^^,^ c	10.18 ± 0.06^b^
pH	7.374 ± 0.016	7.290 ± 0.043	7.330 ± 0.034	7.302 ± 0.021	7.342 ± 0.022
PO2 (mmHg)	76.000 ± 7.180	87.800 ± 3.425	78.910 ± 3.339	83.100 ± 4.433	80.116 ± 2.557
SaO2 (%)	92.00 ± 1.826^a^	85.400 ± 5.435	88.19 ± 4.003^b^	87.376 ± 2.981^b^	89.200 ± 2.440^b^
PCO2 (mmHg)	45.600 ± 2.904	57.580 ± 5.435	52.051 ± 3.006	49.123 ± 2.334	51.270 ± 3.557
Respiratory amplitude (mV)	34.45 ± 10.33^a^	1.35 ± 0.26	11.07 ± 0.78^b^	6.73 ± 1.22^b^^,^ c	10.12 ± 0.34^b^

a P < 0.05, compared with sham group.

b P < 0.05, compared with model group.

c P < 0.05 compared with LMWH group.

So, the treatment effect of Bre (i.p.) at a dosage of 1 mg/kg/day was remarkable. There was a dose‐dependent effect about Bre intervention. Bre‐low and LMWH almost had the same effect (*P *> 0.05). Bre could maintain normal blood pressure and heart rate, and improve animal oxygen saturation, which was useful for the improvement of PE symptoms.

At the same time, **the D‐dimer concentration was determined** by a blood test to help diagnose thrombosis. The D‐dimer was at the lowest concentration in the sham group than the other four groups. The blood clot was degraded by fibrinolysis, which was also promoted by LMWV. Bre could help the APE rats degrade the blood clot, caused by PS or DVT, which was more apparent at the high dose. Thus, Bre has anti‐coagulation activity. Blood clots with PS are difficult to degrade compared to DVT. The D‐dimer concentration was lower in these rats.

**White blood cell counting** in bronchoalveolar alveolar lavage fluid (BALF) was performed for all experimental rats. Compared with the sham group, the total number of white blood cells increased significantly in the model group. After drug intervention, the number of white blood cells began to decline in the Bre injection group, but not the LMWH group. If total white blood cells were classified, the lymphocytes and macrophages were effector cells compared with neutrophils. The percentage of eosinophils remained the same in each group. LMWH did not cause a significant percentage change for white blood cells compared with the model group.

**Expression of specific inflammatory factors** was detected in this study. The experimental rats were killed 2 days after drug intervention. Then, the right lower lung was obtained and weighed. Each sample was homogenized in lysis buffer and PMSF. Then MCP‐1 and IL‐13 protein concentrations were detected by Western blot, what results were listed in **Table** 2. The IL‐13 and MCP‐1 expression was higher for APE model rats than control rats; the differences were statistically significant (*P *<* *0.05). IL‐13 and MCP‐1 expression was lower in the Bre and LMWH intervention groups than the model group, which suggested that Bre could reduce the expression of the target protein. Furthermore, this regulatory effect has a significant dose‐dependent effect. When Bre was injected i.p. at a high dose, the effect was significantly better than LMWH at 48 h. And the high‐dose Bre group had better results than the LMWH group on the regulation of APE with respect to MCP‐1 expression caused by DVT.

**Table 2:** The effects of Bre and LMWH on expression of MCP‐1 and IL‐13 protein in APE rats by Western blot testing (n = 8), Cytokine expression (pg/ml)

	Bre‐low	Bre‐high	LMWH	Model	Sham
MCP‐1	9032 ± 32.33	8109 ± 20.07	11764 ± 39.81	14589.67 ± 15.20	3510 ± 63.17
IL‐13	866.27 ± 44.16	845.16 ± 34.51	834.51 ± 29.12	1173.21 ± 712.30	751.39 ± 43.15

In addition, IL‐13 and MCP‐1 mRNA expression was determined by RT‐PCR technology. The expression of IL‐13 and MCP‐1 mRNA was consistent with Western blot testing results.

## CONCLUSION

Bre was shown to down‐regulate IL‐13 expression in the blood and further down‐regulate MCP‐1 expression, which caused alveolar macrophages (AMs) with lower cell activation efficiency. The end result is to reduce inflammation of lung tissues, which facilitates the autologous thrombolytic reaction and reduction in vasoconstriction in rats with PEs. Bre injections have been used >40 years, and may be recommended for the treatment of PE. Additional clinical trials and data are warranted.

## FUNDING

This study is supported by the Innovation team in Yunnan Province (2015HC025); Yunnan Applied Basic Research, Kunming Medical University Joint Special Research Project (2017FE468(‐061)).

## CONFLICT OF INTEREST

None.
